# Coarse bubble mixing in anoxic zone greatly stimulates nitrous oxide emissions from biological nitrogen removal process

**DOI:** 10.1016/j.wroa.2024.100263

**Published:** 2024-10-06

**Authors:** Haoran Duan, Shane Watt, Dirk Erler, Huijuan Li, Zhiyao Wang, Min Zheng, Shihu Hu, Liu Ye, Zhiguo Yuan

**Affiliations:** a Australian Centre for Water and Environmental Biotechnology (ACWEB, formerly AWMC), The University of Queensland, St. Lucia, Queensland 4072, Australia; b School of Chemical Engineering, The University of Queensland, St. Lucia, Queensland 4072, Australia; c Water Research Centre, School of Civil and Environmental Engineering, University of New South Wales, Sydney, New South Wales 2052, Australia; d Faculty of Science and Engineering, Southern Cross University, Lismore, New South Wales 2480, Australia; e School of Energy and Environment, City University of Hong Kong, Hong Kong SAR, China; f State Key Laboratory of Marine Pollution, City University of Hong Kong, Hong Kong SAR, China

**Keywords:** Nitrous Oxide, N_2_O, Coarse bubble mixing, Denitrification inhibition, Nitrite shunt

## Abstract

The biological nitrogen removal process in wastewater treatment inevitably produces nitrous oxide (N_2_O), a potent greenhouse gas. Coarse bubble mixing is widely employed in wastewater treatment processes to mix anoxic tanks; however, its impacts on N_2_O emissions are rarely reported. This study investigates the effects of coarse bubble mixing on N_2_O emissions in a pilot-scale mainstream nitrite shunt reactor over a 50-day steady-state period. Online and offline N_2_O monitoring campaigns show that coarse bubble mixing in the anoxic zones significantly elevates N_2_O emissions, yielding an extremely high emission factor of 15.5 ± 3.5 %. Intensive sampling and isotopic analyses suggest that the elevated emissions are primarily due to the inhibition of the N_2_O denitrification process by oxygen in the anoxic phase introduced by coarse bubbling. Substituting coarse bubble mixing with submersible pump mixing resulted in a substantial reduction of N_2_O emissions, decreasing the emission factor by an order of magnitude to 1.2 ± 0.8 %. The findings reveal that a previously overlooked factor, coarse bubble mixing, can significantly stimulate N_2_O emissions. The use of coarse bubble mixing in anoxic tanks of biological nitrogen removal warrants caution.

## Highlights

• Mixing methods can influence N_2_O emissions in wastewater treatment processes.
• Coarse bubble mixing significantly increases N_2_O generations in anoxic zones.
• N_2_O emission factors rose to 15.5 % due to oxygen inhibition of N_2_O denitrification.
• Replacing coarse bubble mixing with submersible pump greatly reduced N_2_O emissions.

## 1 Introduction

The biological nitrogen removal process in wastewater treatment inevitably produces Nitrous Oxide (N_2_O), a potent greenhouse gas (GHG) with a global warming potential 273 times that of CO_2_ and a long atmospheric lifetime of 116 years (Edenhofer et al., 2014). There are mainly three microbial pathways responsible for N_2_O generation within the biological nitrogen removal process. In nitrification, Ammonia Oxidizing Bacteria (AOB) produce N_2_O as a by-product of NH_2_OH oxidation to NO_2_^−^ (NH_2_OH oxidation pathway), and through the reduction of NO_2_^−^ (Nitrifier denitrification pathway). In denitrification, N_2_O is a mandatory intermediate in the stepwise reduction of NO_3_^−^ to NO_2_^−^, NO, N_2_O, and eventually to N_2_ (Heterotrophic denitrification pathway) (Domingo-Félez and Smets, 2019; Duan et al., 2021). N_2_O emissions can contribute up to 80 % of the total GHG emissions from a wastewater treatment plant (WWTP) (Daelman et al., 2013), underlining their significance for WWTP operations.

Coarse bubble mixing, typically defined as bubbles larger than 2 mm (De Temmerman et al., 2015), is widely employed in wastewater treatment. In conventional activated sludge process, it is often used to facilitate mixing in the anoxic zones (Rosso et al., 2008). While mechanical mixers are more prevalent, coarse bubble aeration has proven, particularly for large facilities, to be a cost-effective method of providing mixing with limited impact on denitrification in anoxic zones (Eddy et al., 2013). Moreover, coarse bubble mixing has found increased application in recent years with the rising adoption of novel biofilm-based wastewater treatment processes. In moving bed biofilm reactors (MBBR) or integrated fixed-film activated sludge system (IFAS), coarse bubble mixing keeps carriers suspended within anoxic zones (Sander et al., 2016; Yuan et al., 2021; Zheng et al., 2023). In membrane-aerated biofilm reactors (MABR), coarse bubble aeration serves dual purposes of facilitate mixing and scouring the biofilm, contributing to the control and optimization of MABR performance (Bunse et al., 2020; Mehrabi et al., 2020; Silveira et al., 2022).

Despite being a commonly applied technology, the role of coarse bubble mixing in anoxic zones on N_2_O emissions has rarely been reported. In this study, we investigate the impacts of coarse bubble mixing on N_2_O emissions in a pilot-scale mainstream nitrite shunt reactor, operated in steady-state, over a period of 50 days. Isotopic techniques were carried out to reveal the underlying N_2_O generation and consumption mechanisms. This study cautions the use of coarse bubble mixing in anoxic zones.

## 2 Results and discussions

### 2.1 High N_2_O emissions with coarse bubbling

**Fig. 1:**
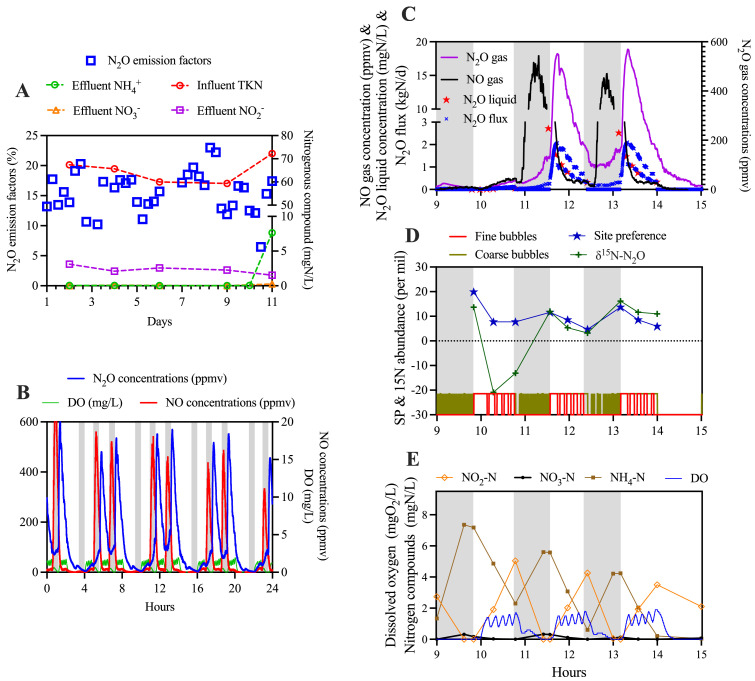
(A) N_2_O emission factors (per 6-hour cycle) in day 1–11. (B) Typical dynamics of N_2_O concentrations in the off-gas monitored on Day 4, showing regular peaks of N_2_O and NO concentrations; Intensive sampling during a typical cycle of SBR operations on day 4: (C) N_2_O/NO emissions and N_2_O liquid concentrations; (D) ^15^N abundances in N_2_O (δ^15^N-N_2_O) and site-preference (SP) of ^15^N in N_2_O; (E) Nitrogenous compounds transformation and dissolved oxygen levels during the operation. Shaded areas indicate the anoxic periods.

By the time N_2_O monitoring commenced, the pilot-scale mainstream nitrite shunt reactor under study had been operated steadily for over 200 days. This 2m^3^ sequencing batch reactor treated real domestic sewage in 6-hour cycles, including three anoxic-aerobic sub-cycles with wastewater feeding in all three anoxic period, settling, and decanting, with a hydraulic retention time of 24 h. A satisfactory total nitrogen removal efficiency of 94.8 ± 0.8 % was achieved via the nitrite pathway. The effluent contained low levels of ammonium (0.1 ± 0.1 mgN/L) and nitrate (0.1 ± 0.1 mgN/L), with nitrite being the predominant nitrogen form at 2.5 ± 0.7 mgN/L (Fig. 1A) (Duan et al., 2022). Note the elevated ammonium concentration in the reactor observed on day 11 was due to a malfunction in the air blower.

Staggeringly high N_2_O emissions were consistently measured immediately following the commencement of on-line N_2_O monitoring. The mean N_2_O emission factor (EF) in the first 11 days was 15.5 ± 3.5 % (mean ± standard deviation), with instances of elevated N_2_O EFs exceeding 22 % (Fig. 1A). In comparison, similar systems have been reported to have EFs in the range of 1.5 % to 3.3 % (Duan et al., 2019; Zhu et al., 2023). Within an operational cycle, high N_2_O peaks consistently occurred during the second and third aerobic periods (Fig. 1B). Notably, regular NO peaks were also observed during the second and third anoxic phases.

To investigate the potential causes of the elevated N_2_O emissions, intensive sampling campaigns were conducted on day 4 (Fig. 1C-E). The high N_2_O peaks exhibited no correlation with the in-reactor concentrations of ammonium, nitrite, or nitrate (Fig. 1E). Instead, the results suggested that N_2_O peaks were likely attributable to the accumulation of N_2_O during the anoxic phase. At the end of the preceding second or third anoxic phase, liquid N_2_O concentrations could accumulate to levels exceeding 2.8 mgN/L, representing over 50 % of nitrite reduced (Fig. 1C). Subsequent aeration during the aerobic phase stripped the accumulated liquid N_2_O, resulting in the observed peak emissions. Given that coarse bubbling was employed during the anoxic phase, it is plausible that the accumulated N_2_O may have been produced from nitrification and/or denitrification processes. Indeed, a recent study reported that coarse bubble aeration in the anaerobic/anoxic zone of a full-scale system can provide oxygen to support nitritation process (Yuan et al., 2021).

To trace the source of the N_2_O generated during the anoxic phase, isotopic N_2_O analyses were conducted. The variations of ^15^N abundances in N_2_O (δ^15^N-N_2_O) and site-preference (SP) of ^15^N in N_2_O on day 4 are presented in Fig. 1D. During the anoxic phase, δ^15^N-N_2_O significantly increased, concurrent with elevated SP values. Conversely, in the subsequent aerobic phase, δ^15^N-N_2_O values decreased in line with aerobic reactions. The observed increase and subsequent decrease in δ^15^N-N_2_O values during the anoxic and aerobic phases, suggest the occurrence of denitrification and nitrification processes, respectively, leading to N_2_O generations (Duan et al., 2017). The correlated increase in δ^15^N-N_2_O and SP values indicates the partial reduction of N_2_O to N_2_, further suggesting denitrification activity during the anoxic phase. However, with only two samples taken in each anoxic phase, it remains unclear whether nitrification may also contribute to N_2_O generation in the anoxic phase.

### 2.2 Source and cause of the high N_2_O accumulations

**Fig. 2:**
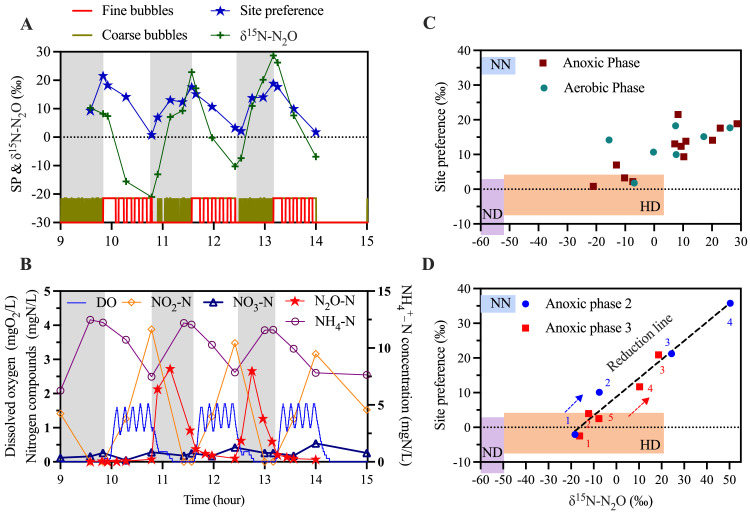
Intensive isotopic sampling on Day 12. (A) δ^15^N-N_2_O and SP values along the SBR cycle; (B) Dissolved nitrogenous compounds and DO variations along the cycle. Grey Shaded areas indicate the anoxic periods; (C) Dual isotope mapping with SP and δ^15^N-N_2_O values; (D) Dual isotope mapping with SP and δ^15^N-N_2_O values in anoxic phase 2 and anoxic phase 3. NN: Hydroxylamine oxidation pathway; ND: Nitrifier denitrification pathway; HD: Heterotrophic denitrification pathway.

To trace the source of N_2_O generations more accurately, a more intensive isotope sampling campaign was conducted on day 12 (Fig. 2A). Consistently, significantly high level of N_2_O accumulations were observed during the anoxic phase of reactor operation. As high as 2.72 mgN/L of N_2_O accumulated in the anoxic phase 2 and 3, while negligible levels of N_2_O accumulation (<0.01 mgN/L) were measured during anoxic phase I (Fig. 2B). A dual isotope mapping approach with SP and δ^15^N-N_2_O was applied (Fig. 2C and D), which is particularly effective for revealing the mixing of N_2_O production pathways and the occurrence of partial N_2_O reduction by heterotrophic denitrification (Gruber et al., 2022; Yu et al., 2020). The increase in SP values during the anoxic phases could be attributed to either the NH_2_OH oxidation (NN) pathway or heterotrophic N_2_O reduction. To distinguish the cause of the SP increase, the fractionation effects of the bulk δ^15^N-N_2_O value offer insight. An increase in δ^15^N-N_2_O indicates the occurrence of nitrification, either via the NN or nitrifier denitrification (ND) pathway, whereas a decrease points to heterotrophic denitrification. Therefore, the simultaneous increase in SP and δ^15^N-N_2_O during the second and third anoxic phases strongly suggests that heterotrophic denitrification was the primary driver of N_2_O production during these periods. Specifically, during the anoxic phase, N_2_O was generated via heterotrophic denitrification and subsequently underwent partial reduction to N_2_, as evidenced by the reduction lines observed in the two anoxic phases (Fig. 2D). The continual reduction line throughout the anoxic phase suggests that partial reduction of N_2_O to N_2_ occurred continuously, indicating incomplete N_2_O reduction, which aligns with the observed N_2_O accumulation at the end of the anoxic phase (Fig. 2B). Negligible mixing effects with N_2_O nitrification generation pathways were observed during anoxic phase. The generation of N_2_O during the anoxic phase was predominantly attributed to incomplete heterotrophic denitrification, rather than nitrification. In addition, during the subsequent aerobic phase, the continuous decrease in δ^15^N-N_2_O points to the activities of nitrification, while the gradual reduction of SP values towards zero suggests a significant contribution of the ND pathway to N_2_O production.

**Fig. 3:**
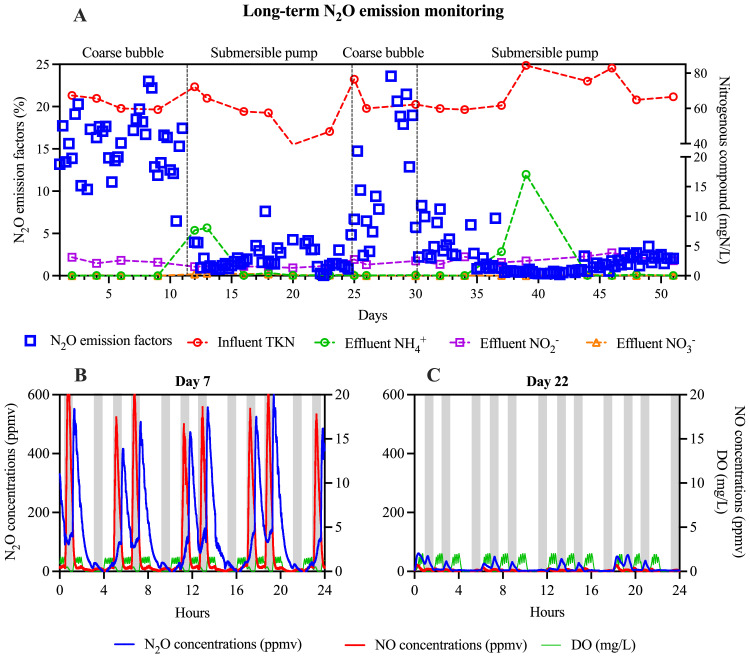
(A) N_2_O emission factors with coarse bubble mixing or submersible pump mixing. Dash line indicated the switch of mixing method in the anoxic phase; (B) Typical dynamics of N_2_O concentrations in the off-gas monitored on Day 7 with coarse bubble mixing; (C) Typical dynamics of N_2_O concentrations in the off-gas monitored on Day 22 with submersible pump mixing. Shaded areas indicate the anoxic periods.

The studied reactor has several unique characters. Firstly, it is a mainstream nitrite shunt reactor characterized by significant accumulations of NO_2_^−^. Prior investigations have highlighted that such NO_2_^−^ accumulations can impede N_2_O reductase activity within denitrification processes, leading to delayed N_2_O reduction (Highton et al., 2022). However, this explanation alone does not account for the absence of N_2_O accumulation in the first anoxic phase. Indeed, past mainstream nitrite shunt studies did not report significant N_2_O accumulation during anoxic denitrification.

Secondly, the pilot reactor implemented coarse bubble mixing during anoxic phase. The application of coarse bubble mixing could conceivably stimulate N_2_O emissions through two avenues: a) coarse bubble aeration can reduce the availability of COD for denitrification; b) introduction of oxygen via coarse bubble mixing may inhibit the anoxic reduction of N_2_O. The coarse bubble aeration inevitably introduced air into the system, which will oxidize the COD in the system. The overall mass transfer coefficient (kLa) for the coarse bubble mixing system was determined to be 1.34 ± 0.004 1/h. During one anoxic phase, the estimated quantity of oxygen introduced into the system in total is approximately 0.42 mg per litre of wastewater, which theoretically consumes a maximum of 0.42 mgCOD per litre of wastewater during this period. In comparison with the total COD in the influent (472.8 ± 85.2 mgCOD/L), the potential consumption of COD by the coarse bubble aeration is negligible. It is unlikely that the coarse bubble aeration will affect denitrification due to reduced COD availability.

It is more likely that oxygen introduced by coarse bubble mixing inhibited the N_2_O reduction process. In the stepwise denitrification processes, N_2_O reductase is most sensitive to oxygen inhibition (Bonin and Gilewicz, 1991; Bonin et al., 2002). Although the DO level was too low to be measured by the DO sensor, coarse bubble aeration might have introduced enough oxygen to inhibit the denitrification process, especially N_2_O reduction. The impaired N_2_O reduction process by coarse bubble aeration led to the accumulation of N_2_O towards the end of the anoxic phase, subsequently being stripped out during the following aerobic phase. This also explains why high emissions were not observed in the first anoxic phase. During the operations, additional readily biodegradable COD became available in the first anoxic cycle due to sludge fermentation (resulting from settling and decanting steps in SBR operations), potentially mitigating the inhibitory effects on N_2_O reduction by being oxidized with the introduced oxygen from coarse bubble mixing. To verify the hypothetical role of coarse bubble mixing, it was replaced by a submersible mixing pump in the reactor operation.

### 2.3 Reduced N_2_O emissions without coarse bubbling

The stop of coarse bubble mixing led to an immediate and substantial reduction in N_2_O emissions, as shown in Fig. 3. Specifically, N_2_O EFs plummeted nearly an order of magnitude from 15.5 ± 3.5 % (*m* ± *s*.d., day 1–11) to 1.9 ± 1.6 % (*m* ± *s*.d., day 12–24). The decline in N_2_O emissions was accompanied by notable reductions in NO emissions throughout the operational period. The coarse bubble mixing was reactivated from day 25 to 30 for reconfirmation, resulting in a temporary resurgence of N_2_O emissions. However, upon restoring the submersible pump mixing, consistently low N_2_O emissions were recorded from day 38 to 51, with an average EF of 1.2 ± 0.8 % (*m* ± *s*.d.). The repeated comparisons between operation periods with and without coarse bubble mixing unequivocally indicate that the presence of coarse bubble mixing was causally linked to the observed high N_2_O emissions in the studied reactor.

**Fig. 4:**
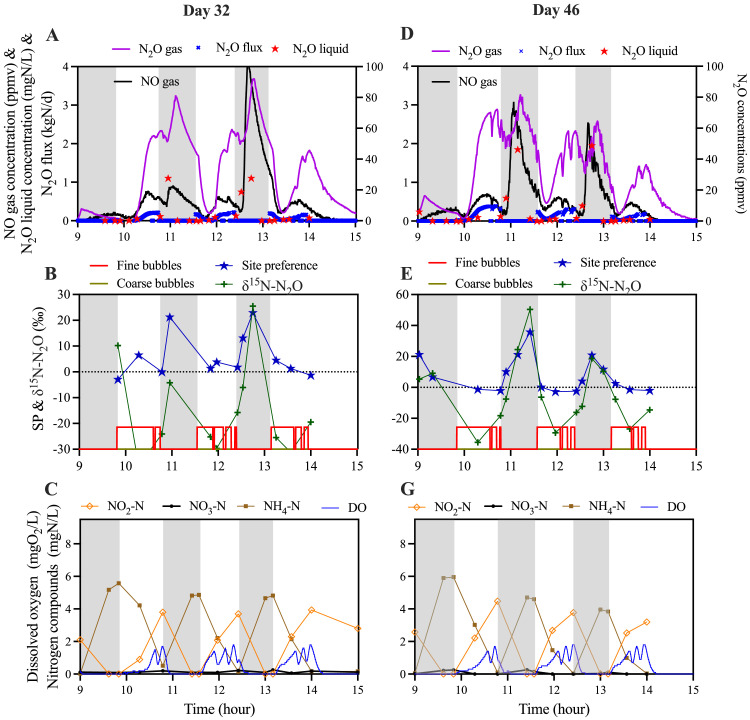
Intensive sampling during a typical cycle of SBR operations on day 32 and day 46 without coarse bubble mixing. (A&D) N_2_O/NO emissions and N_2_O liquid concentrations; (B&E) ^15^N abundances in N_2_O (δ^15^N-N_2_O) and site-preference (SP) of ^15^N in N_2_O; (C&G) Nitrogenous compounds transformation and dissolved oxygen levels during the operation. Shaded areas indicate the anoxic periods.

The intensive sampling campaigns conducted during the operation with the submersible pump provided a clear insight into the system dynamics (Fig. 4). In fact, switching off coarse bubbling did not avoid the accumulation of N_2_O during anoxic reactions. For example, on Day 32 and 46, N_2_O accumulated to levels as high as 2.0 mgN/L during the anoxic phase, a magnitude comparable to that observed during periods with coarse bubble mixing (Fig. 4A and D). However, unlike the instances with coarse bubbling, where N_2_O accumulation often exceeded 2 mgN/L at the end of the anoxic phase (Fig. 1E), the accumulated N_2_O was largely consumed by the conclusion of the anoxic reactions. Correlated increase of SP values during anoxic phase indicated the occurrence of denitrification (Fig. 4B and E). It is noteworthy that the accumulation and subsequent consumption of N_2_O during the anoxic phase closely mirrored the behaviour of NO. Specifically, NO also exhibited accumulation before being gradually consumed towards the conclusion of the anoxic phase, displaying a significant correlation with N_2_O concentrations. The accumulations of NO and N_2_O indicated remaining inhibitions on denitrification. It is likely the DO inhibition on denitrification has not fully recovered in the experimental period.

When aerobic phase starts, SP values quickly dropped and stabilized around 0 ‰. The observed SP values indicated that its major source is nitrifier denitrification (Fig. 4B and E). This is also supported by the observed correlation of N_2_O emission, and nitrite accumulation levels during aerobic phase (Fig. 4C and G). Apparently, replacing coarse bubbling with submersible pump, the accumulated N_2_O during anoxic zone can be largely reduced. The major source of N_2_O emissions became the nitrifier denitrification by AOB during aerobic phase, which was likely stimulated by the accumulation of nitrite, consistent with previous literature (Duan et al., 2018; Ma et al., 2017; Zhao et al., 2023). The results indicate that, without coarse bubble mixing, N_2_O emissions in the nitrite shunt reactor are within the lower range for similar nitrite shunt systems, with emission factors between 1.5 % and 3.3 % (Duan et al., 2019; Zhu et al., 2023).

Coarse bubble aeration system is commonly applied in wastewater treatment. Ideally, mechanical mixers are used in the anoxic zones, but for large plants coarse bubble aeration has proven to be a cost-effective option. The coarse bubble aeration system applied in the pilot-scale system (kLa = 1.34 1/h) is comparable to typical coarse bubbling aeration systems (Chern and Yang, 2003). The experimental results of the pilot-scale reactor operation in this work conclusively showed that the implementation of coarse bubbling can greatly promote N_2_O emissions due to impaired denitrification. As such, careful consideration is required when employing coarse bubble mixing in anoxic zones. Although the findings suggest that switching to non-bubbling mixing strategies may mitigate the associated risks, alternative approaches such as reducing aeration intensity or oxygen transfer efficiency could also potentially alleviate the inhibition of N_2_O reduction and contribute to N_2_O mitigation. Therefore, it is suggested for plants with coarse bubbling should measure their N_2_O emissions and optimize the mixing method when necessary.

## 3 Conclusions

This study demonstrates that coarse bubble mixing in anoxic zones of a pilot-scale mainstream nitrite shunt reactor substantially increases N_2_O emissions, with extremely high emission factors of 15.5 ± 3.5 % measured, due to the inhibition of the N_2_O denitrification process by introduced oxygen. Intensive sampling and isotopic analyses confirmed that this mixing method leads to significant N_2_O accumulation during denitrification in anoxic phases, which is subsequently released during aerobic phases. Replacing coarse bubble mixing with submersible pump mixing reduced N_2_O emissions by nearly an order of magnitude to 1.2 ± 0.8 %, highlighting the significance of mixing methods on N_2_O emissions. Given the identified risks, the plants employing coarse bubble mixing are suggested to monitor their N_2_O emissions and optimize the mixing method when necessary.

## 4 Material and methods

### 4.1 Pilot-scale mainstream nitrite shunt system

This study was carried out in a pilot-scale nitrite shunt system treating real domestic sewage. The nitrite shunt system received step-feed in sequential batch mode. Specifically, the reactor was fed with 2 m^3^ sewage every day in 6 h cycles, each cycle comprising first anoxic feeding, anoxic reaction, first aerobic reaction, second anoxic feeding, anoxic reaction, second aerobic reaction, third anoxic feeding, anoxic reaction, third aerobic reaction, settling, and decanting periods, resulting in an HRT of 24 h. The sludge retention time was maintained at 15 days. The DO concentrations in all three aerobic phases were controlled between 1.0 to 1.5 mgO_2_/L. The pilot-system received Free Nitrous Acid (FNA)-based sludge treatment on a regular basis, which is a commonly applied approach to control the nitrite oxidizing bacteria growth (Duan et al., 2020; Su et al., 2023). Briefly, approximately 25 % of the reactor sludge was collected from the reactor per day, at the end of settling period. This sludge was transferred into a treatment tank to be exposed to a high level of FNA (1.6 - 1.9 mgN/L) for the inactivation of NOB in the system. A detailed description of this pilot system and wastewater characteristics can be found in Duan et al. (2022). While the nitrite shunt was achieved using FNA treatment, the FNA treatment was stopped before the N_2_O monitoring commenced.

### 4.2 N_2_O/NO monitoring and calculation

The N_2_O/NO emissions were monitored online for over 50 days during the stable operation period of the pilot-scale nitrite shunt reactor (day 732–783 of pilot reactor operation). The off-gas of the pilot-scale reactor is collected and sent to a gas conditioner (Horiba VS-5003). The gas conditioner removes the excess moisture and dust from the gas stream before sending it into the analyser. The off-gas N_2_O/NO concentration was continuously monitored by the gas analyser (Horiba VA-5000), with a N_2_O measuring range of 0–500 ppmv and NO measuring range of 0–100 ppmv. The analyser achieves repeatability of ± 0.5 % and linearity of ± 0.1 %. The analyser was calibrated regularly according to the manufacture instructions. N_2_O/NO concentrations were automatically logged every 10 s. A flowmeter is installed at the aeration lines to measure the flowrates of the fine bubbles and coarse bubbles.

The N_2_O emissions were calculated based on the online-monitored results as N_2_O—N emitted= ∑ (C_N2O__—__N, gas_ * Q_f_ * Δt), where C_N2O__—__N, gas_ is the N_2_O—N concentration in the off-gas (mg N_2_O—N/L, converted from ppmv, corrected for temperature); Q_f_ represents the aeration flow rate (L/hr); Δt represents the time interval by which the off-gas N_2_O concentration was measured by the N_2_O analyser. The N_2_O emission factor (EF) is defined as N_2_O—N emitted / TKN loading × 100 %. The EFs in this study were calculated per 6-hour SBR cycle.

### 4.3 Aeration coefficient measurement

Clean water tests were carried out to measure the mass transfer coefficients of the coarse bubble and fine bubble systems in this work (ASCE, 2007). Firstly, reagent grade sodium sulfite (Na_2_SO_3_) was added to deoxygenate the water. The addition was made in excess to reduce the dissolved oxygen to zero. The aeration system was then turned on. During the test, an online handhold DO meter (YSI Prosolo) was used to continuously monitor the DO concentration. The online monitored DO data was fitted using software AQUASIM 2.1d to determine the kLa (Reichert, 1994). Parameter estimation and uncertainty evaluation were carried out in accordance with Batstone et al. (2003).

### 4.4 Analytical methods

For the measurement of NH_4_^+^, NO_2_^−^ and NO_3_^−^ concentrations, filtration was performed first using a 0.45 µm membrane filter (Millipore, Millex GP); and the filtered samples were then analyzed with a Lachat Quik-Chem 8000 Flow Injection Analyzer (Lachat Instrument, Milwaukee, Wisconsin), with the detection limit of 0.002, 0.003 and 0.003 mg N/L for NH_4_^+^, NO_2_^−^ and NO_3_^−^, respectively. For liquid N_2_O concentration determination, the liquid samples collected from the pilot-scale system were immediately filtered through 0.45 um filters and were injected into vacuumed Labco^Ⓡ^ Exetainers. After equilibration of gas and liquid phases, the N_2_O concentrations in the gas phase of the tubes were measured using a Shimadzu GC-9A gas chromatograph (GC) equipped with a micro-electron capture detector (ECD) and a flame ionization detector (FID), respectively. Each Labco Exetainer tube was weighed before and after sampling to determine the sample volume collected. This volume, along with the known total volume of the Exetainers, enables to calculate dissolved N_2_O contained in the original wastewater sample (Sturm et al., 2015).

For the isotopic analysis, liquid samples (10 mL) were taken from the pilot-scale system and immediately filtered through 0.22 um membrane filter (Millipore, Millex GP). Filtered samples were injected by needle syringes into 20 mL glass septa sealed bottles that had been purged with helium. The δ^15^N–N_2_O, and isotopomer (δ^15^Nα, and δ^15^Nβ) signatures of the N_2_O in the headspace of the sample vials were measured on a Thermo Fisher GasBench II interfaced to a Thermo Delta V Plus IRMS following helium sparging (60 mL min^−1^) and cryogenic trapping on a custom-built purge and trap unit. More details can be found in our previous study (Duan et al., 2018).

## CRediT authorship contribution statement

**Haoran Duan:** Writing – review & editing, Writing – original draft, Methodology, Investigation, Formal analysis, Data curation, Conceptualization. **Shane Watt:** Investigation, Formal analysis. **Dirk Erler:** Investigation, Formal analysis. **Huijuan Li:** Investigation, Formal analysis. **Zhiyao Wang:** Investigation, Formal analysis. **Min Zheng:** Investigation, Formal analysis. **Shihu Hu:** Project administration, Investigation, Formal analysis. **Liu Ye:** Investigation, Formal analysis. **Zhiguo Yuan:** Writing – review & editing, Supervision, Project administration, Funding acquisition, Conceptualization.

## Declaration of competing interest

The authors declare that they have no known competing financial interests or personal relationships that could have appeared to influence the work reported in this paper.

## Data Availability

Data will be made available on request
